# Isolation and characterization of novel phages for control of the phytopathogen *Pseudomonas marginalis*

**DOI:** 10.1007/s00253-025-13657-1

**Published:** 2025-12-19

**Authors:** Dina Gamal El-Sayed, Ashraf Fathy Abd El-Rahman, El-Shaimaa Mostafa Abd El-Hamed, Marwa N. Ahmed, Rasha Samir Mohamed

**Affiliations:** 1 Department of Agricultural Microbiology, Faculty of Agriculture, Cairo University, Giza, 12613 Egypt; 2 Bacterial Diseases Research Department, Plant Pathology Research Institute, Agricultural Research Center (ARC), Giza, 12619 Egypt; 3 School of Biotechnology, Nile University, Giza, 12677 Egypt

**Keywords:** Phage therapy, *Pseudomonas marginalis*, Biocontrol, Soft rot disease

## Abstract

**Abstract:**

The current study provides the first detailed characterization of two novel bacteriophages, DG23 and RG24, that infect *Pseudomonas marginalis*, a causative agent of soft rot in potato and other vegetable crops. The phages were assessed for environmental stability, genetic characteristics, and biocontrol efficacy. Both DG23 and RG24 showed broad tolerance throughout a wide pH range (3–9), with RG24 still viable at pH 11, while DG23 was more sensitive to extreme pH conditions. Thermal stability assay demonstrated that both phages remained infectious up to 45 °C, but activity decreased dramatically at higher temperatures, with total inactivation at 75 °C. Phage viability reduced considerably under UV irradiation (254 nm), with DG23 demonstrating better resistance than RG24. Whole-genome sequencing revealed that both phages are lytic, with no integrase, pathogenicity, or antibiotic resistance genes, ensuring biosafety for prospective agricultural uses. Comparative genomic analysis indicated a 99% average nucleotide identity (ANI) between DG23 and RG24, showing they are the same species, but both were genetically distinct from their nearest relative, *Pseudomonas* phage XD2 (ANI 92%). In addition, comparative proteomic and phylogenetic analyses revealed that DG23 and RG24 form a distinct clade within the class *Caudoviricetes*, separate from other related phages. Biocontrol experiments showed that both phages efficiently inhibited potato soft rot when used individually, but when combined, disease severity was decreased by more than 80%, demonstrating the higher efficiency of phage cocktails. These data suggest that DG23 and RG24 are promising, safe, and effective candidates for phage-based biocontrol of soft rot caused by* P. marginalis*.

**Key points:**

• *Novel phages DG23 and RG24 lyse Pseudomonas marginalis and lack virulence genes.*

• *Phages show stability under broad pH, temperature, and UV conditions.*

• *Cocktail treatment reduces potato soft rot severity by more than 80%.*

**Supplementary information:**

The online version contains supplementary material available at 10.1007/s00253-025-13657-1.

## Introduction

Potato (*Solanum tuberosum*) is one of the world’s most important food crops, ranking fifth in economic significance. Therefore, potato plays an important role in food security (Zhang et al. 2024). Losses of up to 30% of total yield may occur during cultivation and postharvest storage (Gustavsson et al. 2011). Bacterial soft rot is one of the most devastating diseases in agricultural production. Multiple rot-causing pectinolytic bacteria are responsible for this disease, e.g., *Pectobacterium*, *Dickeya*, *Pseudomonas*, *Bacillus*, *Burkholderia*, *Pantoea*, *Enterobacter*, *Klebsiella*, *Leuconostoc*, and *Clostridium* (Charkowski 2018; Zhang et al. 2022; Radke et al. 2024). These pathogens can adapt easily and spread in the absence of clear symptoms via infected seeds, contaminated tools, irrigation water, and other equipment used on a farm. Soft rot usually infects vegetable and ornamental fleshy storage organs, e.g., potatoes, carrots, onions, and irises (Perfileva et al. 2025). It causes waterlogged lesions and tissue deterioration that can easily disseminate under favorable conditions (Maciag et al. 2024). Fruity crops like tomato and cucumber are also highly susceptible, especially during handling and storage after harvesting. It may also infect parts above the ground like stems and leaves, destroying crops like cabbage, lettuce, celery, and spinach (Williamson-Benavides and Dhingra 2021). In tropical climates, soft rot often appears on the fleshy stems of bananas, maize, and cassava while these plants are still in the field (Agrios 2005; Sharma and Chauhan 2025). *Pseudomonas marginalis* is known to cause bacterial head rot and soft rot in vegetative tissues, particularly in potato tubers and various leafy or fleshy vegetables such as lettuce, celery, and ornamentals, leading to 30–100% loss of the crop (Dart et al. 2023; Yenjerappa et al. 2025). *P. marginalis* is a pectinolytic bacterium that produces enzymes that lyse the plant cell wall’s pectin, causing rapid tissue softening and collapse. *P. marginalis* is a postharvest disease of varied vegetables and fruits but also infects some crops before harvest (Membré and Burlot 1994; Liao et al. 1997; Ghasemi et al. 2024).

To control bacterial soft rot in crops, chemical approaches are mainly employed, often using agricultural antibiotics and copper-based compounds (Qi et al. 2022). Among these, streptomycin is one of the most often used antibiotics for controlling soft rot (Sang et al. 2015; Shen et al. 2025). Antibiotics are frequently used to treat soft rot disease, but the overuse of these drugs can cause resistance in the bacteria that cause it to evolve, making control less effective, contaminating ecosystems, and perhaps endangering human health, including the risk of cancer (El-Zaemey et al. 2013; Kaur and Garg 2014; Rangasamy et al. 2018; Muteeb et al. 2023). Agronomic practices such as crop rotation with non-host species have been implemented as supplementary control strategies; however, these methods often provide only partial and inconsistent protection, especially under optimal conditions for disease development (Lee et al. 2021). These limitations have led to growing interest in biological control approaches, which present a more sustainable, environmentally responsible, and potentially longer-lasting solution for managing bacterial soft rot.

Among biological control methods, bacteriophages—viruses that specifically infect bacteria and lyse them—have gained considerable attention as possible biocontrol agents. Phages can be classified as lytic, lysogenic, or chronic based on their life cycles. In contrast to their lysogenic and chronic counterparts, lytic phages only undergo a lytic cycle, which causes the host bacteria to be directly lysed without the risk of compromising the host’s pathogenicity (Farooq et al. 2022). Therefore, lytic phages are excellent candidates for biocontrol treatments. Numerous bacterial plant diseases have been found to be successfully controlled by phages (Kering et al. 2019; Choudhary et al. 2025). Recent advances in isolating phages, sequencing genomes, and product formulation have led to commercial phage-based solutions targeting several plant bacterial pathogens. Lytic phages have been isolated from soil in Egypt and shown to have great biocontrol potential against the soft rot-causing pathogen *Pectobacterium carotovorum* (Elhalag et al. 2024).

However, using phages in agriculture comes with challenges. Their limited host range often requires using mixtures of phages (cocktails) for broad control, and bacteria can develop resistance over time. Phages are also vulnerable to environmental factors like UV light, extreme temperatures, and drying out. Despite these challenges, when included in overall disease management strategies, phages have shown great potential for controlling bacterial diseases in crops (Balogh et al. 2010; Thi et al. 2025).

To our knowledge, no bacteriophage targeting *P. marginalis* has been reported. Therefore, this study presents the first isolation, biological characterization, and genomic analysis of two novel *P. marginalis* phages—DG23 and RG24—and assesses their potential as biological control agents against soft rot disease.

## Materials and methods

### Bacterial isolates

The bacterial isolate *P*. *marginalis* LPm33 was previously isolated from a potato tuber showing soft rot symptoms and was provided for use in this study (AbdEl-Hamed 2019). Other bacterial isolates, including plant pathogenic and non-pathogenic strains from various genera, were obtained from the Bacterial Diseases Research Department at the Plant Pathology Research Institute, Agricultural Research Center (ARC) and from the Agricultural Microbiology Department, Faculty of Agriculture, Cairo University, Giza, Egypt.

### Preparation of bacterial suspensions

Bacterial isolates were streaked onto tryptic soy (TS) agar (17 g tryptone, 3 g peptone, 5 g NaCl, 2.5 g dextrose, 2.5 g K_2_HPO_4_, 20 g agar, distilled water to 1 L, and pH 7.2) medium and incubated at 28 °C for 24 h. A single colony was then transferred to 100 mL of TS broth and incubated overnight at 28 °C with shaking at 150 rpm. The culture was then diluted in sterile TS broth to obtain the desired bacterial concentration for subsequent experiments.

### Confirmation of *P. marginalis* identification

#### BIOLOG GEN III MicroPlate system

The BIOLOG GEN III MicroPlate system (Biolog Inc., Hayward, CA, USA) was employed for the identification of the *P*. *marginalis* LPm33 isolate, following protocol A as recommended by the manufacturer. The isolate was initially cultured on TS agar at 28 °C. After a 24-h incubation period, bacterial colonies were collected using sterile cotton swabs and suspended in inoculating fluid A (Catalog No.72401-IF-A) until the desired turbidity level was achieved. Subsequently, 100 μL of the prepared bacterial suspension was dispensed into each well of the GEN III microplate. The inoculated plates were incubated at 30 °C for 24 h, after which results were manually interpreted and analyzed using the MicroLog M/5.2.01 35 software (Biolog Inc. 2001) to identify the bacterial strain.

#### 16S rRNA gene sequencing and phylogenetic analysis

The DNA template of *P*. *marginalis* LPm33 was prepared according to the method followed by Amer et al. (2024). PCR amplification of the 16S rRNA gene was performed using universal primers 27 F (5′-AGAGTTTGATCATGGCTCAG-3′) as the forward and 1492R (5′-GGTTACCTTGTTACGACTT-3′) as the reverse primer (Frank et al. 2008). Approximately 100 ng of DNA template was added to a 50 μL reaction mixture containing 25 µL PCR reaction mixture (amaROnePCR, GeneDi-reX, Miaoli County, Taiwan), 2 μL (0.4 μM) of each primer, and molecular-grade water to complete the volume. The PCR conditions were: 2 min at 94 °C, 35 cycles (1 min at 94 °C, 1.5 min at 55 °C, and 1 min at 72 °C), and 72 °C for 3 min. The PCR product was purified and sequenced using the reverse primer (1492R) by the Sanger sequencing method at MACROGEN (Seoul, Korea). The obtained sequence was compared with the National Center for Biotechnology Information (NCBI) database (Altschul et al. 1990) through the BLAST (basic local alignment search tool) search tool to obtain closely related sequences. Sequence alignment was performed using the MUSCLE program (Edgar 2004), and a phylogenetic tree was constructed based on evolutionary distances using the neighbor-joining method with the software MEGA12 (Kumar et al. 2024). The 16S rRNA gene sequence of the *P*. *marginalis* LPm33 was submitted to the NCBI GenBank database, and the accession number PV875390 was obtained.

### Bacteriophage isolation

Ten *Cucumis sativus* (cucumber) and ten *Cucurbita pepo* (summer zucchini) vegetables were purchased from the local market in Giza governorate in September 2023 for the isolation of bacteriophage infecting *P. marginalis* LPm33. Each vegetable type was rinsed with 500 mL sterile water in a sterile glass beaker. The rinse water was then collected and filtered using a 0.45 µm filter (Millipore, Merck, Darmstadt, Germany). 10 mL of each sample and 5 mL of an overnight *P. marginalis* LPm33 culture were added to 50 mL of TS broth and incubated with shaking at 30 °C. After 24 h incubation, bacterial cultures were spun down for 15 min at 6000 × g; the supernatants were filtered using a syringe filter 0.45 µm and tested for phage infectivity by spot test (Kropinski et al. 2009; El-Sayed et al. 2022). Briefly, 5 µL of the filtrate was spotted onto a TS agar layer inoculated with *P. marginalis* LPm33. The formation of plaques on the bacterial lawn was considered as evidence of the presence of the target lytic phages. The two phages DG23 and RG24 are available at the Microbiology Lab, Agricultural Microbiology Department, Faculty of Agriculture, Cairo University, 12613, Giza, Egypt.

### Purification of bacteriophages

Phage lysates were purified using the plaque assay method as described by Adams (1959). Briefly, serial dilutions of the phage suspension were prepared in phosphate-buffered saline (PBS; 8 g/L NaCl, 0.2 g/L KCl, 1.44 g/L Na_2_HPO_4_, and 0.24 g/L KH_2_PO_4_, pH 7.4). For each dilution, 0.5 mL was mixed with 0.5 mL of an overnight culture of *P. marginalis* LPm33 and overlaid with 5 mL of semi-solid TS agar (0.7% w/v) on top of a solidified TS agar base layer (2% w/v). Plates were incubated at 30 °C for 24 h. A single well-isolated plaque was picked and inoculated into 5 mL of an overnight culture of *P. marginalis* LPm33, followed by incubation with shaking at 30 °C for 24 h. The culture was clarified by centrifugation at 6000 × g for 15 min, and the supernatant containing phage particles was filtered through a 0.45 µm syringe filter. The purification procedure was repeated thrice to ensure homogeneity of the phage lysate.

### Transmission electron microscopy (TEM)

The phage lysates were deposited on an ultrathin carbon film coated lacey carbon supported copper grid (Sigma-Aldrich, Taufkirchen, Germany), and then negatively stained with 2% (w/v) phosphotungstate at pH 7.2. After complete air drying, the stained grids were examined using an electron microscope (JEM-2100 HRT high-resolution TEM, Jeol, Japan) at the Electron Microscopy Unit, National Research Center, Giza, Egypt.

### Phage host range determination

The host range of phages was determined using a spot assay, with some modifications (Kropinski et al. 2009) to determine the lytic activity of isolated phages against bacterial isolates. Briefly, 10 µL of phage suspension containing approximately 10^8^ plaque forming units per milliliter (PFU/mL) was spotted onto the bacterial lawn. A clear zone was recorded as a positive result (+), indicating bacterial lysis, while the absence of clearing was considered negative (-). Isolates that showed positive lysis in the spot test were further evaluated by determining the efficiency of plating (EOP) using a double-layer agar (DLA) assay. A 500 µL aliquot of bacterial suspension, adjusted to an optical density at 600 nm (OD_600_) of 0.2, corresponding to approximately 1.6 × 10^8^ colony-forming units per milliliter (CFU/mL), was mixed with 500 µL of phage suspension containing approximately 10^8^ PFU/mL. EOP was calculated as the ratio of the phage titer on the test strain to that on the primary host strain. The activity of phages was categorized as high (EOP > 0.1), moderate (0.005 < EOP < 0.099), low (EOP < 0.005), or inexistent (no plaques detected), according to Green et al. (2017). Detailed information on the bacterial strains evaluated in the host range analysis of phages DG23 and RG24 is presented in Supplemental Table S1.

### Environmental stability of isolated phages

The stability of *Pseudomonas* phages DG23 and RG24 was evaluated under different environmental stress conditions, including ultraviolet (UV) radiation exposure, pH, and temperature.

For pH stability, 1 mL of phage suspension (10^7^ PFU/mL) was added to 9 mL of TS broth adjusted to various pH values (2–12) and incubated at 30 °C for 1 h. A sample at pH 7 served as the control. For thermal stability, phage suspensions (10^7^ PFU/mL) were incubated at different temperatures (25, 40, 45, 55, and 70 °C) for 1 h. For UV stability, 5 mL of phage lysate was placed in sterile 9-mm Petri dishes and exposed to 254-nm UV at an energy dose of 15 Joules using a Bio-Link BLX-E254 UV crosslinker (Vilber Lourmat, Collégien, France). The phages were irradiated for 30 and 60 min, respectively. Untreated phage lysate was used as a control. In all assays, surviving phages were quantified using the DLA method after incubation at 30 °C for 24 h.

### Genome sequencing and analysis

Phage genomic DNA was extracted from 200 μL of sucrose cushion–concentrated phage suspension using the GeneJET™ Viral DNA and RNA Purification Kit (Thermo Fisher Scientific, Waltham, MA, USA) following the manufacturer’s protocol. DNA concentration was quantified with a Qubit™ 3 Fluorometer (Thermo Fisher Scientific, Waltham, MA, USA) using the Qubit™ dsDNA HS Assay Kit (Thermo Fisher Scientific, Waltham, MA, USA). The DNA was prepared for sequencing using the Illumina TruSeq DNA Nano kit (Illumina, San Diego, CA, USA) and sequenced on an Illumina MiSeq platform (50× sequencing coverage) (El-Sayed et al. 2022).

The assembled contigs were generated from raw sequencing following standard quality control and trimming steps using Fastp (Chen et al. 2018). Assembly was performed using CLC genomic workbench version 25.0.2 (QIAGEN, Aarhus, Denmark). To identify viral sequences, assembled contigs were analyzed using CheckV (Nayfach et al. 2021). These tools enabled robust viral classification based on genome features and hallmark gene content. Two independent methods were used to assess viral genome completeness. The first method is an AAI-based completeness assessment in which amino acid identity (AAI) values were determined by aligning translated contigs to a curated viral genome database using PHASTER (PHAge Search Tool Enhanced Release) (Arndt et al. 2016). For each contig, the top hit identity score (ID), expected genome length, completeness percentage, confidence level, and the number of matching protein-coding genes were documented. The second approach involved HMM-based completeness, where hidden Markov model (HMM) profiles from the CheckV marker gene database were used to identify conserved viral protein domains. The number of HMM hits and predicted completeness range (lower and upper bounds) were recorded. Each viral contig was phylogenetically classified based on AAI alignment results to the NCBI database. For each sequence, the top AAI hit, percent identity, alignment fraction (AF%), and confidence score were recorded, allowing taxonomic inference and evaluation of relatedness to known viruses. To further validate viral origin, *k*-mer frequency analysis was performed using Jellyfish with a *k*-mer size of 31. This helped characterize compositional similarity patterns between assembled contigs and known viral reference genomes.

### Genome annotation

The assembled contigs of both phage genomes were functionally annotated using two complementary approaches to ensure annotation accuracy and completeness. Prokka version 1.2.0 (Seemann 2014) was employed for genome annotation, predicting coding sequences (CDSs), tRNAs, rRNAs, and assigning putative functions based on curated databases such as UniProt and Pfam. In parallel, PHASTEST (PHAge Search Tool with Enhanced Sequence Translation) (Wishart et al. 2023), a specialized bacteriophage annotation and identification platform, was used to refine gene predictions, identify prophage-related features, and classify predicted proteins into functional categories. Functional annotation was also conducted using the blastp tool in the NCBI non-redundant protein database. Antimicrobial resistance genes and virulence factors were predicted using the Comprehensive Antibiotic Resistance Database (CARD) (Alcock et al. 2023) and the Virulence Factor Database (VFDB) (Chen et al. 2005), respectively. The genomic maps of both phages were constructed using PhageScope (Wang et al. 2023).

### Comparative genomics

The complete genome sequences of the two phages were compared to publicly available phage genomes using the BLASTn algorithm at NCBI to identify the most closely related reference genomes. Average Nucleotide Identity (ANI) values between the sequenced phages and their closest relatives were calculated using both the JSpeciesWS online service (Richter et al. 2016) and the FastANI tool in Proksee (Grant et al. 2023).

The whole-genome sequences of the two tested phages were deposited into the NCBI GenBank database and identified as *Pseudomonas* phage DG23 and *Pseudomonas* phage RG24, under the accession numbers of PX094395 and PX123149, respectively.

### Comparative proteomics

To investigate the evolutionary relatedness of the isolated phages DG23 and RG24 with other related phages, phylogenetic and proteomic analyses were performed based on the amino acid sequences of the large terminase subunit. The protein sequences were retrieved from the NCBI GenBank database and aligned using ClustalW (Thompson et al. 1994). Phylogenetic trees were constructed using the Neighbor-Joining method with 1000 Bootstrap replicates to evaluate branch support values. Bootstrap values exceeding 75% were represented as bubbles in the resulting tree. The tree was visualized using iTOLv7 (interactive tree of life) (Letunic and Bork 2024).

For the global proteomic comparison, the complete proteomes of DG23 and RG24 were analyzed using ViPTree (Virus Proteomic Tree Server) (Nishimura et al. 2017), which computes genome-wide similarity based on normalized tBLASTx scores between all proteins encoded in the phage genomes. The resulting tree was visualized to determine the placement of DG23 and RG24 among members of the class *Caudoviricetes*. The red-highlighted branch represents the two newly identified phages, while the red star indicates their specific clade within the global proteomic tree.

### Effect of bacteriophages on soft rot induction by *P. marginalis* LPm33 on fresh potato slices

The efficacy of bacteriophages in suppressing soft rot development was assessed according to the method described by Zhang et al. (2024), with modifications. Fresh tubers of potato cv. Spunta were washed, surface sterilized with 70% ethanol, rinsed with sterile distilled water, and sliced into 5 mm-thick discs using a sterile scalpel. The slices were placed individually in sterile Petri dishes lined with moist sterile filter paper. The bacterial suspension was adjusted to 1.6 × 10^8^ CFU/mL. Two bacteriophage suspensions were propagated and prepared in TS broth, achieving final concentrations of 1.6 × 10^8^ PFU/mL for both the individual phage treatments and the combined phage treatment. To minimize residual bacterial debris, phage lysates were clarified by centrifugation at 10,000 × g for 30 min followed by filtration through a 0.2 µm membrane filter (Sartorius, Göttingen, Germany). A superficial well (5 mm) was made using a sterile scalpel in the center of each slide. Each slice was inoculated at the center with 20 µL of the treatment mixture as follows: (1) positive control: 10 µL of bacterial suspension + 10 µL of sterile TS broth, (2) negative control: 20 µL of sterile TS broth, (3) phage DG23 treatment: 10 µL of bacterial suspension + 10 µL of phage DG23 suspension, (4) phage RG24 treatment: 10 µL of bacterial suspension + 10 µL of phage RG24 suspension, and (5) phage combination: 10 µL of bacterial suspension + 10 µL of phage DG23 (5 µL) and phage RG24 (5 µL) suspensions. All mixtures were freshly prepared before use. Each treatment was applied to five replicate slices. The inoculated slices were incubated at 28 °C for 72 h in the dark. After incubation, the area of the soft rot (cm^2^) was measured for each slice using ImageJ software (NIH, Bethesda, MD, USA). Standardized digital photographs were taken for all slices using a high-resolution digital camera under uniform lighting, with a millimeter scale included in each image as a reference. Lesion areas were determined by outlining the visibly rotted regions using the freehand selection tool in ImageJ, followed by calculation of the area based on the reference scale. The inhibitory effect of phage treatments was calculated as a percentage reduction in soft rot area compared to the control group using the formula: Inhibition (%) = [(Ac − At)/Ac] × 100, where Ac is the mean soft rot area in the control treatment, and At is the mean soft rot area in the phage-treated group.

### Statistical analysis

An experiment to evaluate the effect of bacteriophages on soft rot induction was carried out using a completely randomized design with five replicates per treatment. Variance analysis (ANOVA) followed by post hoc pairwise comparisons using Tukey’s Honestly Significant Difference (HSD) test was applied to assess the differences in means between treatments (*P* ≤ 0.05). All analyses were carried out using IBM SPSS Statistics version 25.0 (IBM Corporation, New York, USA, 2017), and figures were constructed using R v4.3.2 (R Core Team 2024).

## Results

### Confirmation of* P. marginalis* identification

The isolate LPm33 has been identified as *P*. *marginalis* through phenotypic and molecular analyses. The BIOLOG GEN III MicroPlate system showed a similarity index of 0.562 to *P*. *marginalis* (Supplemental Fig. S1). The partial 16S rRNA gene sequence also supported this identification, showing 99.16 and 99.06% similarity to *P*. *marginalis* strain LMG 2210 (NR_027230.1) and *P*. *marginalis* strain ATCC 10844 (NR_112072.1) sequences in the NCBI database, respectively. The phylogenetic tree of *P*. *marginalis* LPm33 and closely related isolates based on partial 16S rRNA gene sequences using the neighbor-joining method with 1000 bootstraps is shown in Supplemental Fig. S2.

### Bacteriophage isolation

Two distinct bacteriophages infecting *P. marginalis* were successfully isolated from the rinse water of cucumber and zucchini vegetables. During the initial screening, clear and well-defined plaques were observed on bacterial lawns, indicating lytic activity against the host (Supplemental Fig. S3). The plaques varied in size and morphology, confirming the presence of different phage types. To ensure purity and eliminate the possibility of mixed populations, individual plaques were carefully picked and subjected to at least three consecutive rounds of purification using the DLA assay. This repeated single-plaque isolation yielded purified phage stocks suitable for further characterization.

### Transmission electron microscopy

**Fig. 1:**
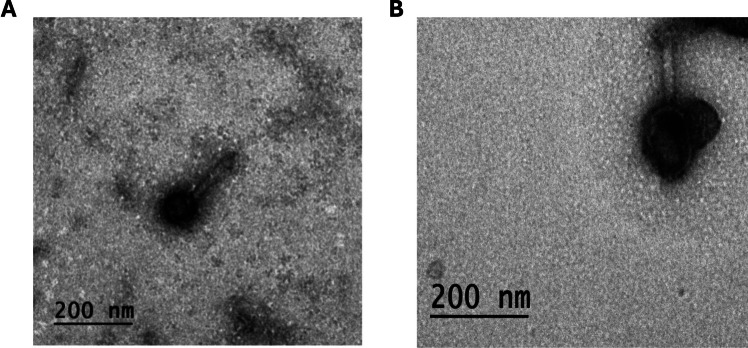
TEM micrographs of *Pseudomonas* phages DG23 and RG24 negatively stained with 2% (w/v) phosphotungstic acid (pH 7.2). **A** Phage DG23 exhibits a hexagonal head (72.6 × 71.46 nm) and a long tail of approximately 148 nm. **B** Phage RG24 displays a larger hexagonal head (128 × 94) and a remarkably long tail of approximately 132 nm. Images were obtained at a direct magnification of 60,000x

TEM revealed distinct morphological characteristics of the two isolated *Pseudomonas* phages. Phage DG23 displayed an icosahedral (hexagonal in appearance) head measuring approximately 72.6 × 71.46 nm, accompanied by a straight, relatively long contractile tail of about 148 nm in length. Phage RG24 exhibited a much larger capsid, with an icosahedral head measuring approximately 128 × 94 nm, and a long contractile tail measuring around 132 nm. This morphology is consistent with a myoviral-like morphotype (Fig. 1).

### Host range of phages DG23 and RG24

The lytic activity of phages DG23 and RG24 against 19 bacterial isolates is shown in Table 1. Testing 19 plant pathogenic and non-pathogenic isolates from five bacterial genera revealed that three isolates of *P*. *marginalis* showed transparent lysis by the phages. The other 16 isolates, belonging to different genera, showed no lysis. Both phages DG23 and RG24 exhibited high infectivity, showing strong lytic activity (EOP > 0.1) against their bacterial host and two *P. marginalis* strains. This indicates that the two phages possess efficient lytic potential within *P. marginalis* isolates.

**Table 1:** Host range analysis of both phages DG23 and RG24 against plant pathogenic and non-pathogenic bacterial isolates

Bacterial strains	Accession No.*	Phage		EOP	
		DG23	RG24	DG23	RG24
*Agrobacterium tumefaciens* strain BAAg4	PP506592	-	-	-	-
*Erwinia amylovora* strain SEa1	OR906985	-	-	-	-
*Pectobacterium atrosepticum* strain MH3C	OR538566	-	-	-	-
*Pectobacterium carotovorum* strain 100H	OP603322	-	-	-	-
*Pseudomonas aeruginosa* strain BPeL6	PP864138	-	-	-	-
*Pseudomonas chlororaphis* strain 13AS_BR13	MW019508	-	-	-	-
*Pseudomonas cichorii* strain Lett7	PP748272	-	-	-	-
*Pseudomonas frederiksbergensis* strain GPlK17	OR197540	-	-	-	-
*Pseudomonas kilonensis* strain GPlK18	OR197541	-	-	-	-
*Pseudomonas marginalis* strain LPm33	PV875390	+	+	1	1
Presumptive *Pseudomonas marginalis* strain LPm37	N/A	+	+	0.99	0.99
Presumptive *Pseudomonas marginalis* strain LPm36	N/A	+	+	0.98	0.96
*Pseudomonas putida* strain GPlL23	OR197545	-	-	-	-
*Pseudomonas vancouverensis* strain BApK9	OR197536	-	-	-	-
*Ralstonia solanacearum* strain H230822	OR533690	-	-	-	-
*Pseudomonas aeruginosa* strain VS05	PQ497619	-	-	-	-
*Pseudomonas fluvialis* strain VS09	PQ497573	-	-	-	-
*Pseudomonas nicosulfuronedens* strain VS15	PQ49762	-	-	-	-
*Pseudomonas sediminis* strain VS19	PQ497628	-	-	-	-

*National Center for Biotechnology Information (NCBI) GenBank accession number; clear lysis (+) and no lysis (-). N/A (not applicable) indicates isolates that were identified based solely on biochemical tests

### Environmental stability of isolated phages

**Fig. 2:**
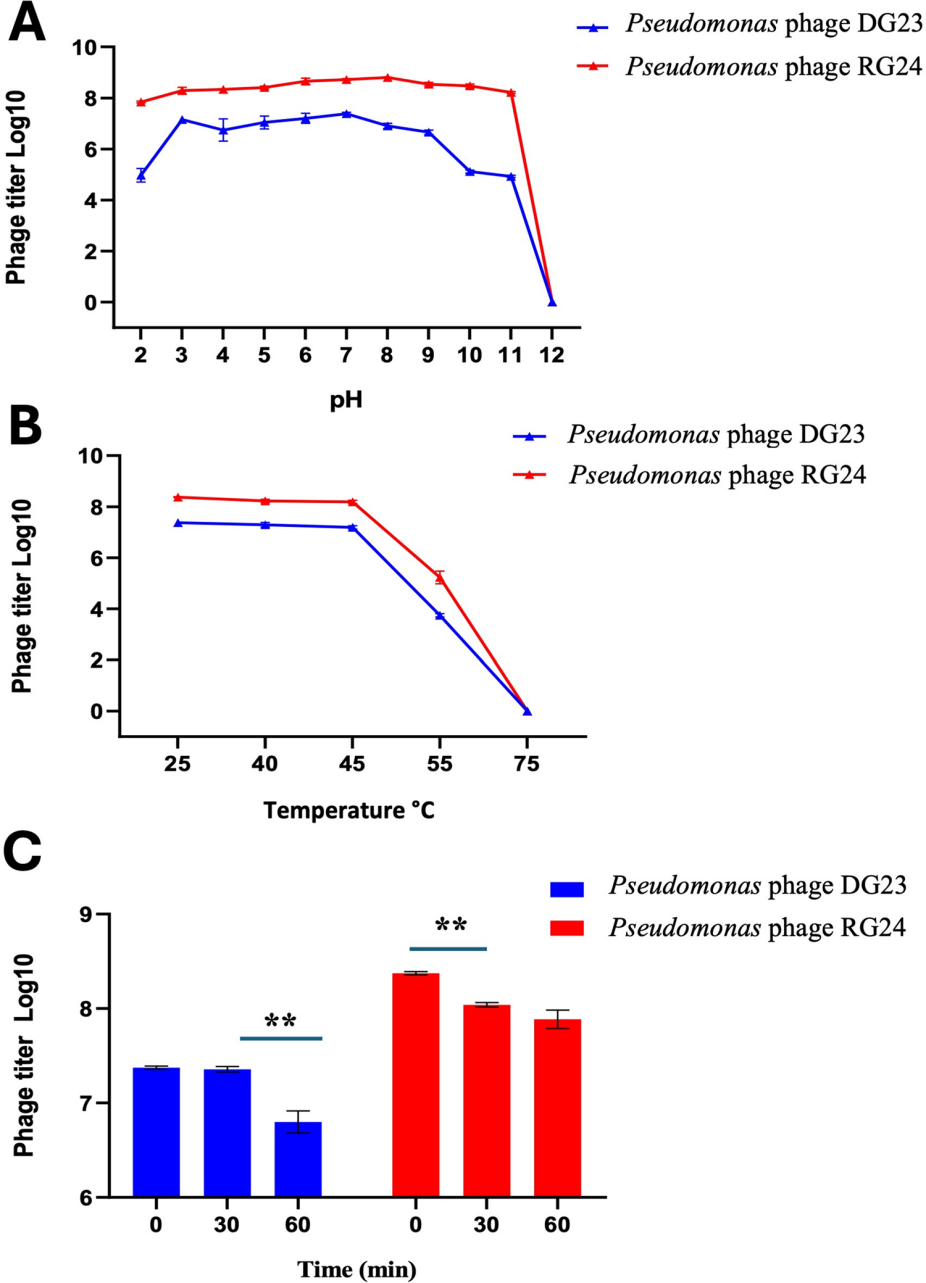
Environmental stability of *Pseudomonas* phages DG23 and RG24. **A** Stability under different pH conditions (2–12) for 1 h. **B** Thermal stability across a range of temperatures (25–75 °C) for 1 h. **C** Stability under UV irradiation at 254 nm over time intervals (0–60 min). Data represent the mean of three independent replicates. Error bars indicate the standard deviation. **P < 0.01; ***P < 0.001; ****P < 0.0001

Temperature, UV radiation, and pH are critical factors influencing the stability and infectivity of bacteriophages. In this study, both DG23 and RG24 demonstrated considerable stability across a broad pH range (2–12) (Fig. 2A). Interestingly, RG24 retained activity even under highly alkaline conditions (up to pH 11), whereas DG23 showed greater sensitivity to both acidic and strongly alkaline environments.

Both phages DG23 and RG24 exhibited similar thermal tolerance patterns (Fig. 2B). They remained stable up to 45 °C, but their activity decreased by approximately 50% at 55 °C, and complete inactivation occurred at 75 °C (*P* < 0.001).

Phage stability under UV radiation was also assessed (Fig. 2C). Specifically, phage DG23 showed no detectable reduction after 30 min of UV exposure, whereas phage RG24 exhibited a slight decrease (*P* < 0.01). However, RG24 demonstrated greater tolerance than DG23 after 60 min of exposure (*P* < 0.01), indicating variation in UV resistance between the two phages.

### Genome characterization

For the *Pseudomonas* phage DG23 genome, a total of 171 genes were annotated in contig 1, which was 94.305 bp in length. The GC content of the genome was 52.44%. Although the precise viral region boundaries could not be explicitly defined, AAI-based estimates indicated an expected genome size of approximately 95.468 bp, suggesting near-complete genome recovery. The AAI-based completeness was calculated at 98.78%, with a high-confidence classification. AAI analysis identified 16 viral protein-coding hits, with the closest match to GCA_000916255.1, showing 58.11% AAI and 58.59% alignment fraction (AF%), and a low AAI error score of 3.54.

**Fig. 3:**
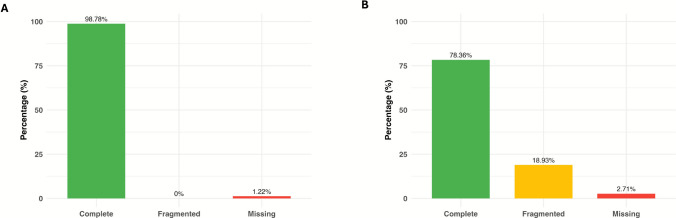
The genome completeness metrics of sequenced phages. **A** *Pseudomonas* phage DG23 **B** *Pseudomonas* phage RG24

Complementary HMM-based analysis further supported these findings, detecting 80 hallmark viral genes and estimating genome completeness between 88.81% and 99.49% (Fig. 3A). Together, the AAI- and HMM-based results demonstrate strong concordance between two independent estimation methods, confirming the high quality and near-completeness of the assembled viral genome.

For the *Pseudomonas* phage RG24 genome, a single contig of 84.579 bp was obtained, containing a total of 146 predicted genes. The GC content of the genome was 48.94%. AAI-based estimates indicated an expected genome size of approximately 95.496 bp, corresponding to an estimated completeness of 88.57% with high-confidence classification. The AAI analysis detected 16 viral protein-coding hits, with the closest match to GCA_002957195.1, showing 60.45% AAI and 61.59% alignment fraction (AF%), and a low AAI error score of 3.54.

HMM-based analysis supported these findings, identifying 71 hallmark viral genes and estimating genome completeness between 78.36% and 97.29% (Fig. 3B). Together, the AAI- and HMM-based results suggest a high-quality genome assembly with near-complete coverage.

**Fig. 4:**
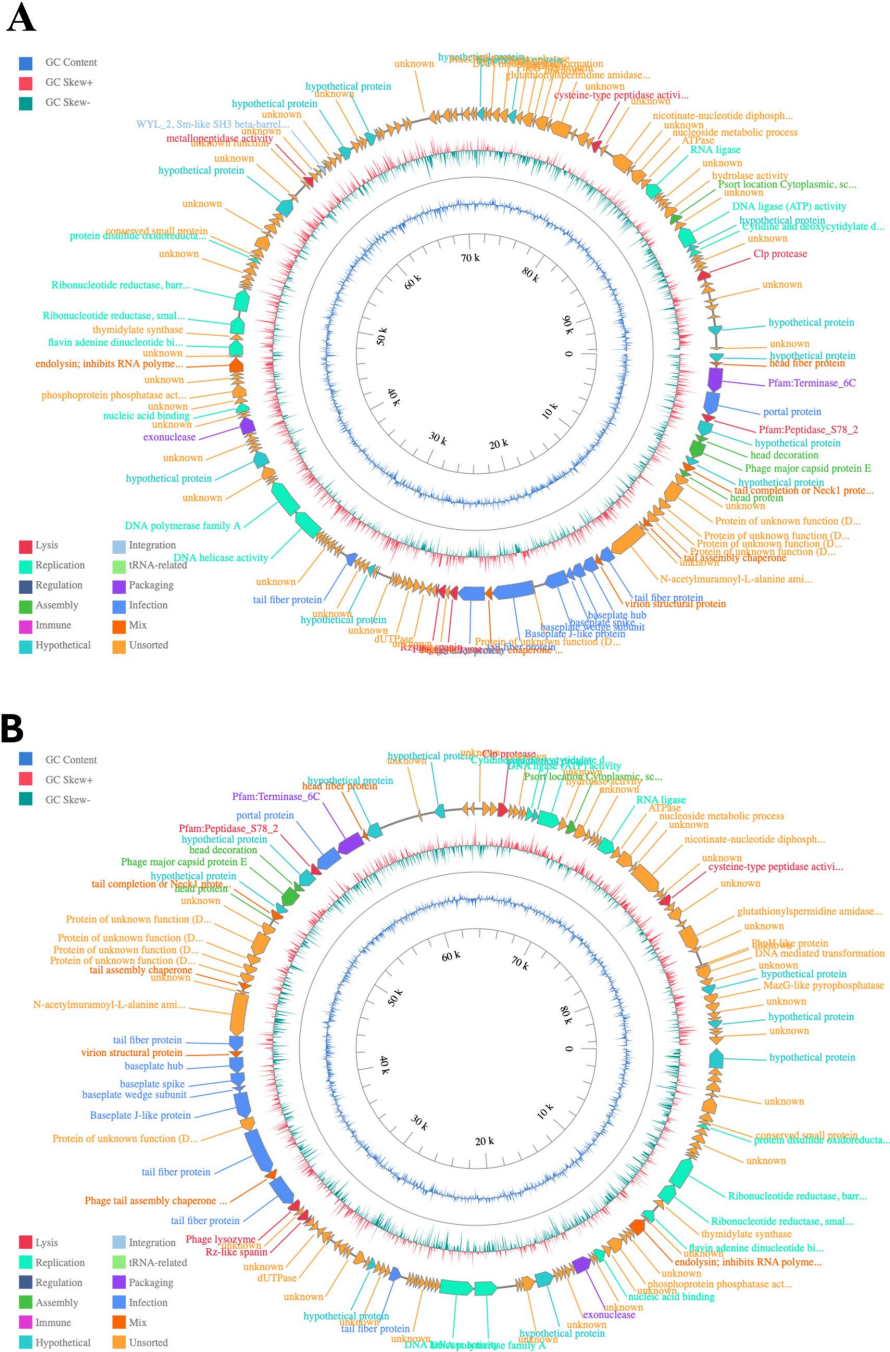
Genome representation of *Pseudomonas* phages DG23 (**A**) and RG24 (**B**). ORFs are shown as colored arrows indicating transcriptional direction, with functional categories represented by distinct colors: lysis (red), replication (green), regulation (yellow), assembly (cyan), immune (purple), hypothetical proteins (light blue), and others as indicated in the legend. The inner rings display GC content (blue) and GC skew (green for positive, purple for negative), highlighting shifts that correspond to replication origins and termini. Gene annotations are labeled around the outer circle, with structural, replication, and lysis modules

The Prokka and PHASTEST annotation analyses revealed that *Pseudomonas* phages DG23 (Fig. 4A) and RG24 (Fig. 4B) have roughly analogous genomic structures. Despite this similarity, noticeable differences in gene arrangement and annotation have been identified. The modular structure of both genomes is maintained, with grouped coding sequences organized into functions such as lysis, replication, regulation, assembly, and hypothetical proteins.

The inner rings of each circular genome map show GC content and GC skew profiles where the alternating positive and negative skew regions exhibit potential replication origins and termini.

**Fig. 5:**
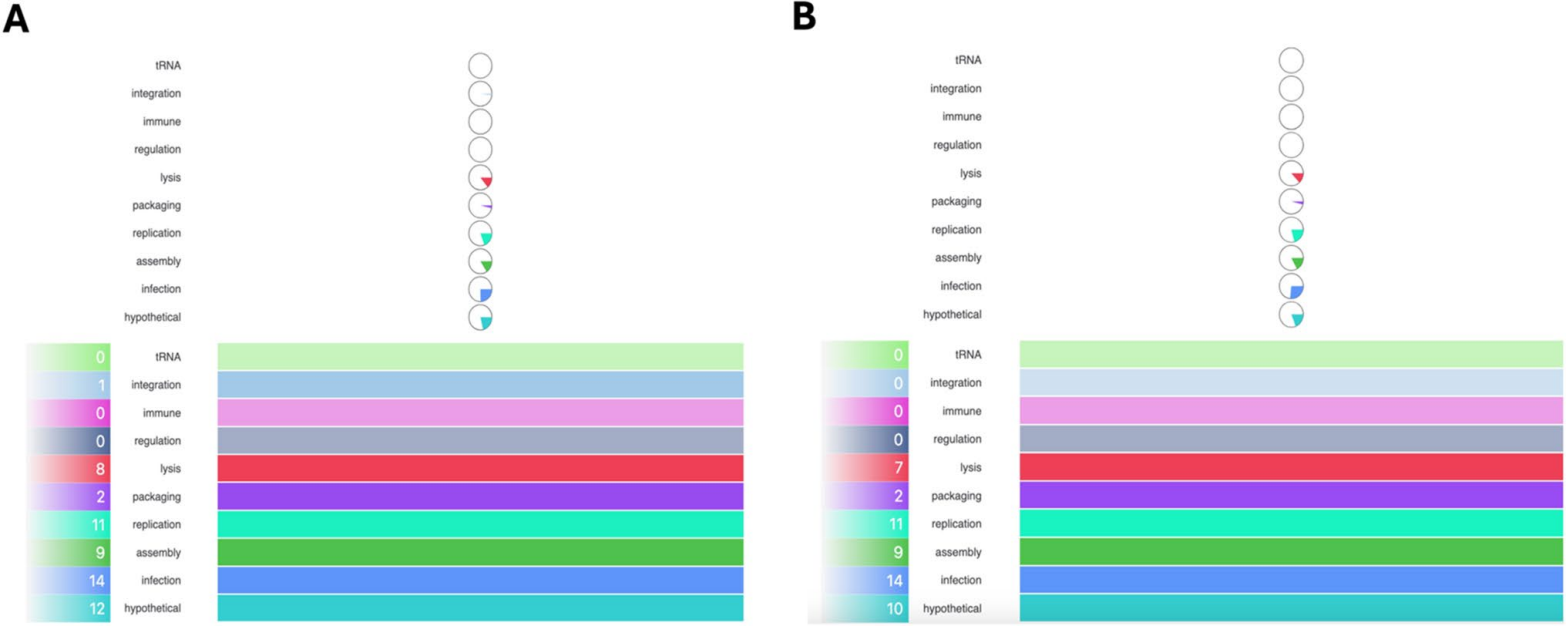
The distribution of predicted gene functions in the genomes of *Pseudomonas* phages **A** DG23 and **B** RG24, as determined by automated annotation pipelines. Functional categories are represented by distinct colors and arranged vertically for clarity

In DG23, we identified 69 predicted coding sequences (CDSs) spread across ten functional groups (Fig. 5A). The largest fraction belonged to hypothetical proteins (12 genes), reflecting the fact that many phage genes remain uncharacterized. Infection-related genes (14 genes) included those for tail fibers, baseplate proteins, and other host-interaction components. Replication genes (11 genes) encoded enzymes such as DNA polymerase, helicases, and proteins for nucleotide metabolism. Structural and assembly genes (9 genes) included those for capsid proteins, terminase subunits, and other parts needed to build new virus particles. The lysis gene cluster, consisting of eight genes, includes those encoding endolysins and holins. We also found two packaging genes, one regulatory gene, and one integration-related gene, but the lack of core integrase domains in encoded proteins suggests DG23 cannot integrate into a host genome. Importantly, no tRNA genes, immune-related genes, antimicrobial resistance genes, or virulence factors were detected.

RG24 harbors a broadly similar set of functions, although the numbers varied slightly. Its genome included 10 hypothetical protein genes, 14 infection-related genes, 11 replication genes, 9 assembly genes, and 7 lysis genes (Fig. 5B). Two packaging genes were also present. Like DG23, RG24 carried no genes for regulation, integration, tRNAs, immune functions, antimicrobial resistance, or virulence factors. The presence of infection, replication, assembly, and lysis modules suggests that RG24 also follows a lytic lifecycle. Detailed open reading frames (ORFs) annotations for both DG23 and RG24 are provided in Supplemental Tables S2 and S3.

NCBI BLASTn analysis was performed to compare the genomes of *Pseudomonas* phages DG23 and RG24 against all bacteriophage sequences available in the NCBI database. The results revealed that both phages showed the highest genomic similarity to *Pseudomonas* phage XD2 (GenBank accession no. PQ288046) (91.7 similarity %), supporting its selection as the closest relative for comparative genomic analysis. Additionally, genome-based similarity analysis and current International Committee on Taxonomy of Viruses (ICTV) guidelines revealed that both phages DG23 and RG24 are assigned to the class *Caudoviricetes* but remain unclassified at the family level (unclassified *Caudoviricetes*).

**Fig. 6:**
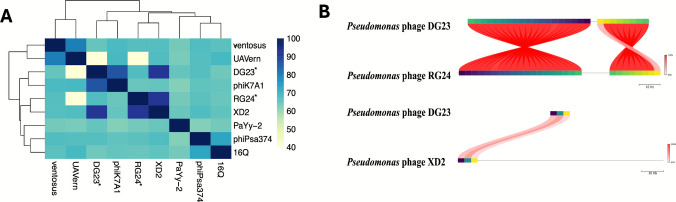
Comparative genomic analysis of isolated *Pseudomonas* phages. **A** Heatmap showing the ANI values among the isolated phages (DG23 and RG24 marked with asterisks) and other *Pseudomonas* genomes. Color intensity represents the degree of genomic similarity, with darker blue shades indicating higher ANI values (up to 100%) and lighter blue shades indicating lower similarity (down to 40%). **B** Whole-genome alignment of *Pseudomonas* phages DG23, RG24, and XD2 (GenBank accession no. PQ288046). Genomes are represented as linear maps, with predicted protein-coding genes shown as colored arrows. Gene colors indicate homologous gene clusters shared among genomes. Ribbons connect homologous regions, with ribbon color intensity representing pairwise nucleotide identity according to the scale shown (range: 92–100%). Crossed ribbons indicate genomic inversions. Genome sizes are indicated by the scale bars (10 kb)

To investigate the genetic relatedness between the sequenced phages, we performed a pairwise ANI% determination of *Pseudomonas* phages DG23, RG24, and other *Pseudomonas* phages (Fig. 6A). There was notable clustering of DG23 and RG24 (99% ANI), which suggests that the two phages share considerable genomic similarity. Although *Pseudomonas* phage XD2 exhibited the highest ANI value (92%) with DG23 and RG24, it branched into a separate cluster, suggesting that XD2 belongs to a different evolutionary lineage. Furthermore, whole-genome alignment (Fig. 6B) demonstrated the presence of large and conserved regions between DG23 and RG24, as indicated by the red connecting blocks. In contrast, phage XD2 exhibited more limited conserved regions with the DG23 genome. These results indicate that DG23 and RG24 are genetically closely related, whereas XD2 is more divergent, supporting their potential classification into distinct phage clusters.

**Fig. 7:**
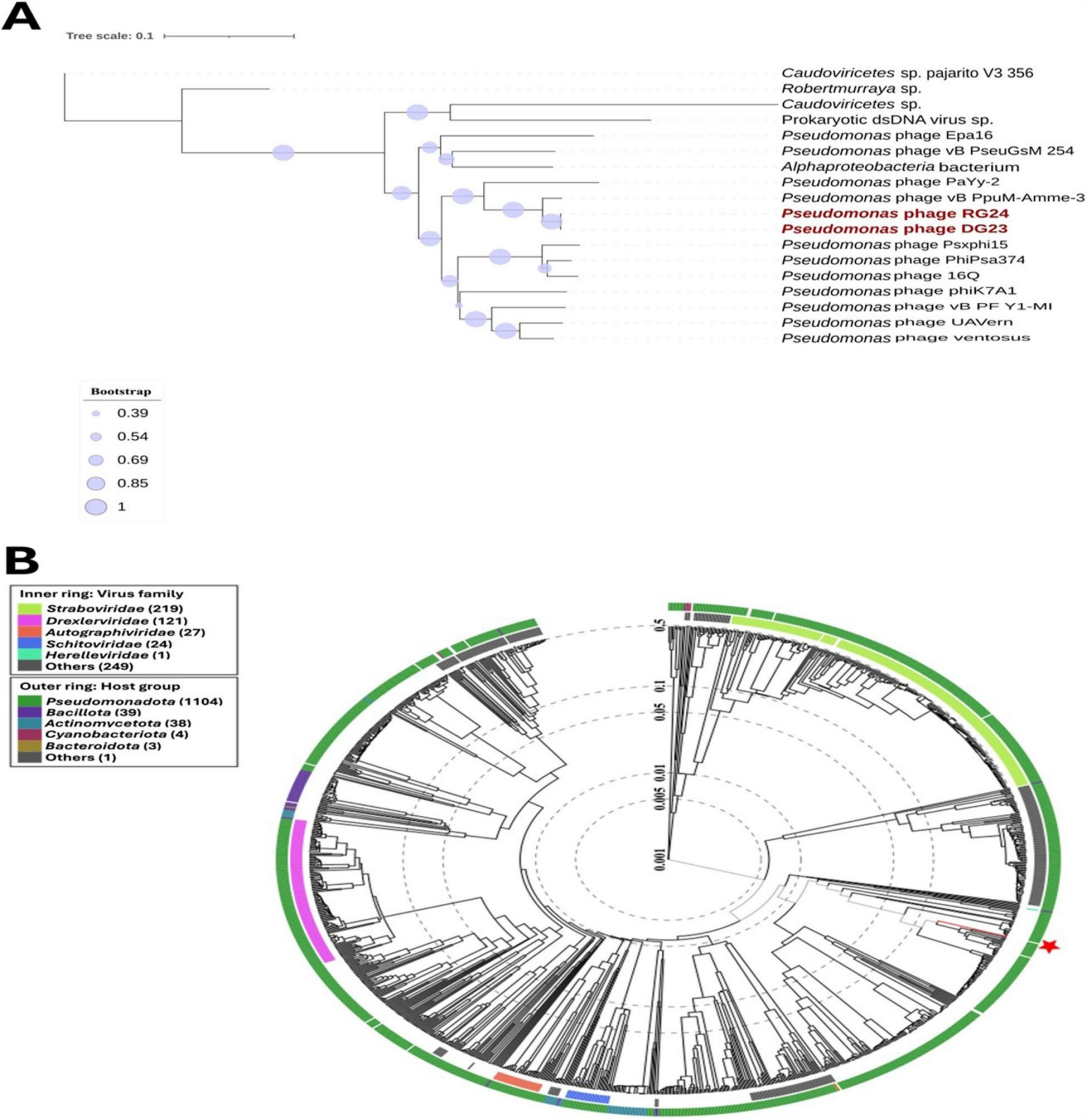
Proteomic analysis of the isolated *Pseudomonas* phages. **A** Phylogenetic tree based on terminase gene sequence comparisons among the *Pseudomonas* phages DG23 and RG24 and other closely related phages. Bootstrap values exceeding 75% (based on 1000 replicates) are represented as bubbles on the tree. **B** Global proteomic tree showing the placement of DG23 and RG24 (red-highlighted branch) among members of the class *Caudoviricetes*. The red star indicates the clade containing DG23 and RG24

To further determine the taxonomic position and evolutionary relationships of the isolated phages, phylogenetic analysis was performed based on the terminase large subunit gene sequences of DG23, RG24, and related phages. The resulting tree revealed that phages DG23 and RG24 clustered closely together, indicating a close genetic relationship and supporting their classification within the same species-level clade (Fig. 7A). Both phages were distantly related to other *Pseudomonas* phages, forming a distinct lineage that reflects their unique genomic characteristics.

Additionally, a global proteomic tree generated using ViPTree demonstrated the overall relationship of DG23 and RG24 with other members of the class *Caudoviricetes* (Fig. 7B). In this analysis, DG23 and RG24 grouped together in a well-defined clade (marked by a red star), separate from other known phages, confirming their close proteomic relatedness yet distinct evolutionary position.

### Effect of bacteriophages on soft rot induction by* P. marginalis *on potato slices

**Fig. 8:**
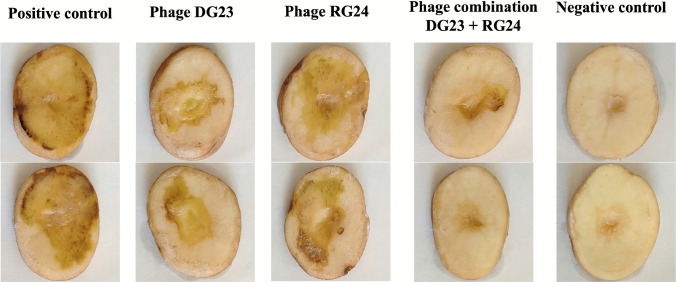
Effect of phages’ treatments on the occurrence of soft rot disease on potato slices inoculated with *P*. *marginalis* strain LPm33

The application of bacteriophages significantly reduced the soft rot induced by *P*. *marginalis* LPm33 on potato slices (Table 2 and Fig. 8). The highest soft rot area was recorded in the positive control group (23.90 ± 2.74 cm^2^), while no disease development was observed in the negative control. The combination of phages DG23 and RG24 resulted in the greatest reduction of soft rot development, with a soft rot area of 4.10 ± 0.81 mm^2^ and the highest inhibition percentage (82.95 ± 1.76%). This was followed by phage DG23 alone, which significantly reduced the soft rot area to 7.88 ± 1.38 mm^2^, corresponding to an inhibition percentage of 67.17 ± 2.14%. Phage RG24 showed a moderate effect, with a soft rot area of 12.88 ± 0.76 mm^2^ and an inhibition percentage of 45.81 ± 3.11%. The combined phage treatment was significantly more effective than either phage used individually.

**Table 2:** Soft rot area (cm^2^) and soft rot inhibition percentage on potato slices infected with *Pseudomonas marginalis* LPm33 under different phage treatments

Treatment	Soft rot area (cm^2^) ± SD	Soft rot inhibition (%) ± SD
Phage DG23	7.88 ± 1.38^c^	67.17 ± 2.14^b^
Phage RG24	12.88 ± 0.76^b^	45.81 ± 3.11^c^
Phage combination (DG23 + RG24)	4.10 ± 0.81^d^	82.95 ± 1.76^a^
Positive control	23.90 ± 2.74^a^	-
Negative control	0.0 ± 0.0^e^	-

Data represent the mean of five replicates, soft rot inhibition (%) = [(Ac − At)/Ac] × 100, where Ac is the average soft rot area in the positive control and At is the average soft rot area in the phage-treated group, SD = standard deviation; means followed by different letters within a column are significantly different according to Tukey’s HSD test at *P* ≤ 0.05.

## Discussion

This study provides the first detailed characterization of two novel bacteriophages, DG23 and RG24, infecting *P. marginalis*. This Gram-negative bacterium produces pectolytic enzyme that causes softness of potato tubers, resulting in total crop loss (Ghasemi et al. 2024)*. P. marginalis* is also a significant postharvest pathogen that causes soft rot in a wide range of harvested fruits and vegetables (Liao et al. 1997). Pectate lyase (pel), levan, and pyoverdine production have been shown to play an important role in the maceration of plant tissue and pathogenicity of *P. marginalis* in soft rot disease (Liao et al.^1997^; Zucker et al. 1972). However, effective and reliable methods to control bacterial soft rot are currently limited (Charkowski 2018). In this study, two bacteriophages capable of infecting *P. marginalis* strain LPm33 were isolated, each producing distinct and clear plaques on bacterial lawns. Additionally, we determined the host ranges of the two isolated phages against 19 different bacterial isolates. The two phages showed only target selectivity against *P. marginalis* strains. These results are in line with previous studies showing that *Pseudomonas* phages exhibit a narrow host range (Celik et al. 2024; Zhang et al. 2024).

Phage stability in the plant growth environment is crucial for field applications. Therefore, the current study also assessed the environmental stability of the isolated two phages. Various internal and external factors in the plant growth environment can alter the stability of phages, hence reducing their efficacy (Orynbayev et al. 2020). Soil and plant surfaces represent complex ecosystems where temperature fluctuations, pH shifts, and UV radiation may severely decrease phage viability (Abdelrhim et al. 2021; Queslati et al. 2022). Both DG23 and RG24 exhibited strong resilience across a wide pH spectrum (3–9), which suggests they can withstand the natural variability encountered in rhizosphere and phyllosphere habitats. Interestingly, RG24 tolerated even more extreme alkalinity (up to pH 11), while DG23 was relatively more sensitive to both acidic and alkaline extremes. These observations align with previous reports that phages are generally stable within moderate pH ranges but often lose infectivity at extremes due to capsid protein denaturation or disruption of lysozyme activity needed for host recognition (Liu et al. 2021; Reimer et al. 2009). Rombouts et al. (2016) investigated the pH tolerance of new phages for the biocontrol of bacterial blight in leek and found that phages were stable from pH 4 to 12 for 24 h. Thermal tolerance assays revealed that both DG23 and RG24 maintained infectivity up to 45 °C but displayed a sharp decline in activity above this threshold. At 55 °C, phage numbers were reduced by half, and at 75 °C, they were completely inactivated. These results are consistent with prior studies where elevated temperatures were shown to extend latency periods and impair replication efficiency (Rombouts et al. 2016). Exposure to UV radiation posed another challenge, as both phages exhibited time-dependent reductions in viability. DG23 was more resistant, maintaining stability under short exposures, whereas RG24 was more sensitive to prolonged UV treatment. This is consistent with previous studies demonstrating the inherent susceptibility of phages to UV-induced DNA damage (Bai et al. 2022; Duarte et al. 2018). Thus, the tested phages are stable throughout a wide range of environmental conditions, including temperatures, pH values, and UV exposure. This corroborates their potentials for biocontrol applications.

Whole-genome sequencing and annotation provided critical insights into the biology and safety profile of DG23 and RG24. Both phages were determined to be lytic, as evidenced by the absence of integrase genes and the presence of genes encoding endolysins, which mediate host cell lysis during the lytic cycle (Kornienko et al. 2021). The lack of integrase genes in *Pseudomonas* phages is indicative of a strictly lytic lifestyle, as these enzymes are essential for genome integration during lysogeny (Carr et al. 2025). Their absence suggests that the phages are unable to establish a lysogenic cycle and instead rely exclusively on host cell lysis for replication. Additionally, no antimicrobial resistance or virulence genes were detected, minimizing risks associated with lysogeny or horizontal gene transfer. Their genome architecture, with clear clusters for replication, structural assembly, and host lysis, is characteristic of lytic phages infecting *Pseudomonas* species.

The relatively large fraction of hypothetical proteins is not unexpected, given that phage genomes often harbor uncharacterized elements that could encode novel proteins with unrecognized roles in infection or host specificity.

Additionally, both phages share a conserved genomic framework, yet they differ in specific gene complements—particularly in hypothetical proteins—which could underpin distinct host interactions and ecological niches. The modular organization and GC skew shifts observed in both genomes support the concept of evolutionary modularity, likely shaped by horizontal gene transfer and recombination events within phage populations infecting *Pseudomonas* species.

Both phages were found to lack the ORFs for tRNAs, implying that they would rely significantly on their hosts for survival and function. Comparative analysis revealed that DG23 and RG24 share nearly identical genome synteny, with an ANI of 99%, suggesting they are closely related variants of the same species. However, both phages were genetically distinct from their nearest relative, *Pseudomonas* phage XD2 (GenBank accession no. PQ288046), with an ANI of 92%. The ANI value below the 95% species divergence threshold further supports the notion that DG23 and RG24 represent novel *Pseudomonas* phages.

The biocontrol efficacy of both DG23 and RG24 phages against potato soft rot has been investigated. When applied individually, both phages significantly reduced disease severity, but the combined treatment provided the highest protection, reducing soft rot development by more than 80%. The superior performance of the cocktail is likely due to complementary host recognition strategies, which reduce the likelihood of bacterial escape through resistant mutants. This phenomenon has been consistently documented in phage therapy studies, where cocktails targeting multiple receptors on bacterial surfaces enhance durability and effectiveness (Farooq et al. 2022). Consistent findings have been reported for phage-based control of *Pectobacterium* and *Dickeya* spp., where cocktail treatments were more effective than single-phage applications (Kering et al. 2019; Vu et al. 2024).

Although DG23 and RG24 phages exhibit high genomic similarity (99% ANI) and share morphological and host range characteristics, combining closely related phages in a cocktail may still offer therapeutic advantages. Previous studies have demonstrated that even phages with very high sequence identity may still differ in their adsorption kinetics, burst size, and latent period, and may also differ in the expression of tail-associated proteins (Defives et al. 1993; Zhang et al. 2024). These phenotypic variations can translate into different infection dynamics, which may impose diverse selective pressure on the bacterial population (Liang et al. 2025). Consequently, this diversity in infection dynamics can prolong the period in which the bacterial population develops resistance with respect to a single phage. The intent of including both phages was also directed to try and demonstrate some synergistic or additive effect in an in vivo setting involving plant tissue, where the environment may alter phage activity in ways that differ from the in vitro conditions.

Overall, our results indicate that DG23 and RG24 not only represent genetically novel *P. marginalis* phages but also demonstrate promising features for use as biocontrol agents. Their ability to survive across broad pH and temperature ranges, their strictly lytic nature, and their effectiveness in reducing soft rot severity strongly support their potential for real-world application.

## Conclusion

This study reports the first isolation and characterization of lytic bacteriophages that infect *P. marginalis*. The two phages, DG23 and RG24, remained stable under various pH, temperature, and UV conditions. Genomic investigations revealed their lytic lifestyle, the absence of virulence or resistance factors, and a conserved modular genome structure. Comparative genomics and proteomics indicated that DG23 and RG24 are closely related yet unique from previously identified *Pseudomonas* phages, making them novel members of this group. Both phages effectively decreased soft rot signs on potato slices, with the cocktail treatment providing the best amount of protection.

These data suggest the safety and efficacy of DG23 and RG24 for long-term *P. marginalis* biocontrol. However, this study was limited to laboratory-scale and in vitro assays. Future studies should involve formulation strategies to improve environmental persistence, evaluation in greenhouse and field conditions, and the preparation of effective phage cocktails.

## Supplementary information

Below is the link to the electronic supplementary material.

ESM 1 (DOCX 1.38 MB)

ESM 2 (XLSX 60.7 KB)

## Data Availability

No datasets were generated or analysed during the current study.
