# Supplementary material for: Isolation and characterization of novel phages for control of the phytopathogen Pseudomonas marginalis
Source: Appl Microbiol Biotechnol. 2025 Dec 19;109(1):276. doi: 10.1007/s00253-025-13657-1 (PMC12718236; doi:10.1007/s00253-025-13657-1)
Supplement: Supplementary file 1 — (DOCX 1.38 MB) [file 253_2025_13657_MOESM1_ESM.docx]

**Supplemental Material**

**Isolation and Characterization of Novel Phages for Control of the Phytopathogen *Pseudomonas marginalis***

**Journal: Applied Microbiology and Biotechnology**

Dina Gamal El-Sayed^1^, Ashraf Fathy Abd El-Rahman^2^, El-Shaimaa Mostafa Abd El-Hamed ^2^, Marwa N. Ahmed^3,1^*, Rasha Samir Mohamed^1^

^1^Department of Agricultural Microbiology, Faculty of Agriculture, Cairo University, Giza 12613, Egypt.

^2^Bacterial Diseases Research Department, Plant Pathology Research Institute, Agricultural Research Center (ARC), Giza 12619, Egypt.

^3^School of Biotechnology, Nile University, Giza, 12677, Egypt.

*Corresponding author:

Marwa N. Ahmed, PhD

Lecturer

School of Biotechnology

Nile University

12677

Giza, Egypt

marwanabil@nu.edu.eg

https://orcid.org/0009-0009-3685-6859


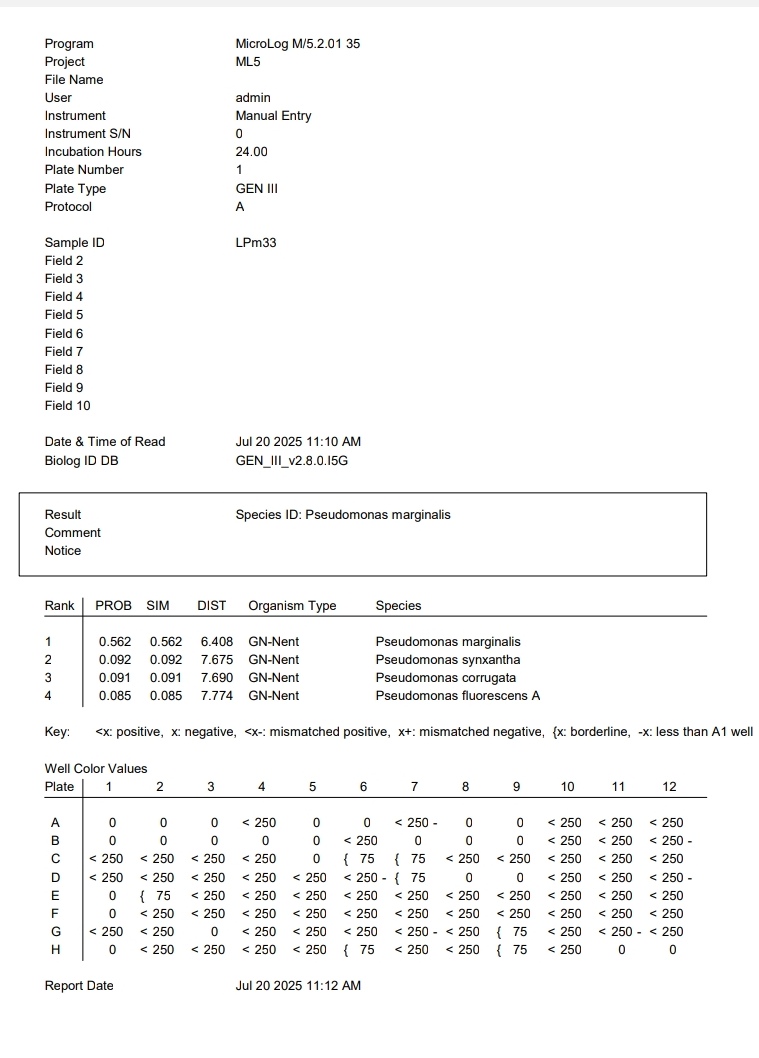


**Fig. S1.** Identification of *P*. *marginalis* LPm33 by the BIOLOG GEN III MicroPlate System.


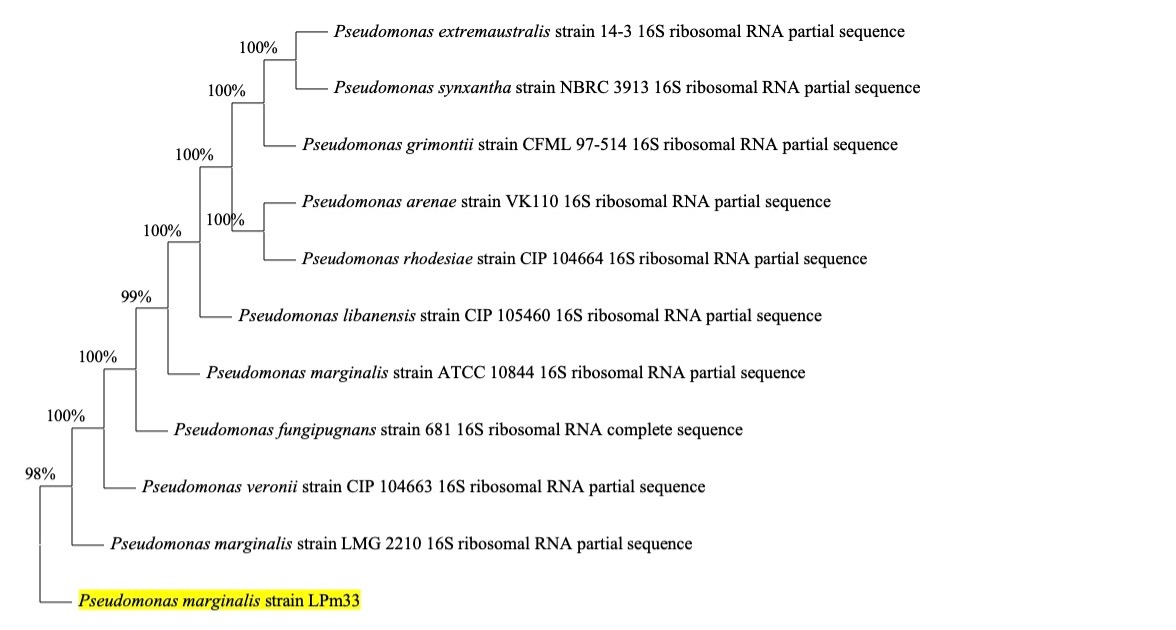


**Fig. S2.** Phylogenetic analysis of *Pseudomonas marginalis* LPm33 and closely related isolates based on 16S rRNA gene sequences using the neighbor-joining method.


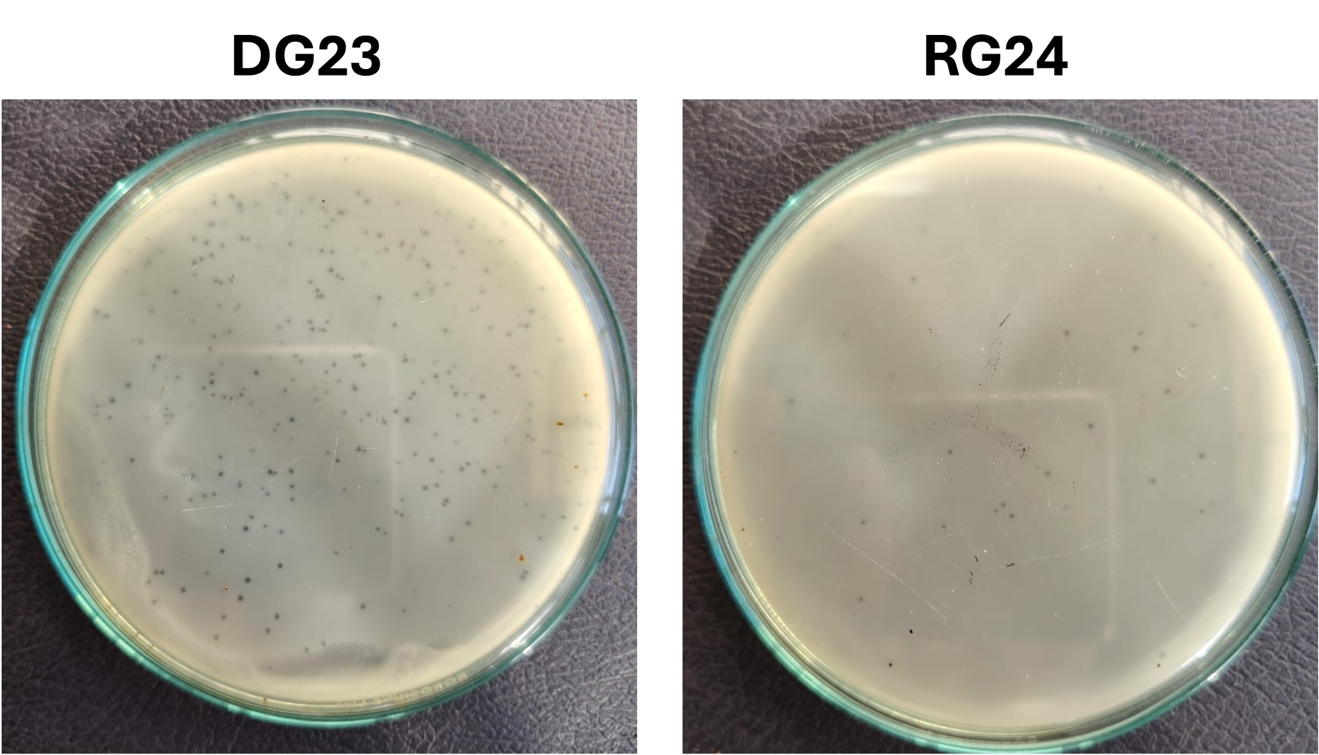


**Fig. S3.** Plaque morphology of Pseudomonas phages DG23 and RG24 on the host strain P. marginalis LPm33.

**Table S1.** Bacterial strains used for host range analysis of the phages DG23 and RG24, including their accession numbers, isolation sources, geographic origins, and year of isolation.

| Bacterial strain | Accession No.* | Isolation source | Geographic location | Isolation year |
| --- | --- | --- | --- | --- |
| *Agrobacterium tumefaciens* strain BAAg4 | PP506592 | Apricot | Beheira, Egypt | 2021 |
| *Erwinia amylovora* strain SEa1 | OR906985 | Pear | Giza, Egypt | 2019 |
| *Pectobacterium atrosepticum* strain MH3C | OR538566 | Potato | Egypt | 2017 |
| *Pectobacterium carotovorum* strain 100H | OP603322 | Potato | Egypt | 2017 |
| *Pseudomonas aeruginosa* strain BPeL6 | PP864138 | Peach rhizosphere soil | Wadi El Natrun, Egypt | 2021 |
| *Pseudomonas chlororaphis* strain 13AS_BR13 | MW019508 | Guava rhizosphere soil | Beheira, Egypt | 2019 |
| *Pseudomonas cichorii* strain Lett7 | PP748272 | Lettuce | Qalyubia, Egypt | 2023 |
| *Pseudomonas frederiksbergensis* strain GPlK17 | OR197540 | Plum rhizosphere soil | Giza, Egypt | 2022 |
| *Pseudomonas kilonensis* strain GPlK18 | OR197541 | Plum rhizosphere soil | Giza, Egypt | 2022 |
| *Pseudomonas marginalis* strain LPm33 | PV875390 | Potato tuber | Behira, Egypt | 2014 |
| Presumptive *Pseudomonas marginalis* strain LPm37 | N/A | Potato tuber | Shark El Awainat, Egypt | 2015 |
| Presumptive *Pseudomonas marginalis* strain LPm36 | N/A | Potato tuber | Gharbia, Egypt | 2014 |
| *Pseudomonas putida* strain GPlL23 | OR197545 | Plum rhizosphere soil | Giza, Egypt | 2022 |
| *Pseudomonas vancouverensis* strain BApK9 | OR197536 | Apricot rhizosphere soil | Sadat City, Egypt | 2021 |
| *Ralstonia solanacearum* strain H230822 | OR533690 | Potato | Egypt | 2017 |
| *Pseudomonas aeruginosa* strain *VS02* | OR197545 | Poultry farm | Giza, Egypt | 2024 |
| *Pseudomonas khazarica*strain *VS06* | OR197536 | Poultry farm | Giza, Egypt | 2024 |
| *Pseudomonas nicosulfuronedens* strain VS10 | OR533690 | Poultry farm | Giza, Egypt | 2024 |
| *Pseudomonas sediminis* strain VS08 | PQ497619 | Poultry farm | Giza, Egypt | 2024 |

*National Center for Biotechnology Information (NCBI) GenBank accession number; clear lysis (+) and no lysis (-). N/A indicates isolates that were identified based solely on biochemical tests.
